# Viewing Time Behavior in a Diverse Sample of 320 Pedohebephilic and Teleiophilic Men Who Have Committed Child Sexual Offense and Who Have Not

**DOI:** 10.1007/s10508-025-03286-0

**Published:** 2025-12-26

**Authors:** Charlotte Hoffmann, Christopher Sinke, Till Amelung, Klaus M. Beier, Henrik Walter, Inka Ristow, Martin Walter, Kolja Schiltz, Boris Schiffer, Jonas Kneer, Tillmann H. C. Krueger

**Affiliations:** 1 Department of Psychiatry, Social Psychiatry and Psychotherapy, Division of Clinical Psychology and Sexual Medicine, Hannover Medical School, Carl-Neuberg-Str. 1, 30625 Hannover, Germany; 2 Institute of Sexology and Sexual Medicine, Charité-Universitätsmedizin Berlin, Corporate Member of Freie Universität Berlin, Humboldt-Universität Zu Berlin, and Berlin Institute of Health, Berlin, Germany; 3 MSB Medicalschool Berlin, Hochschule für Gesundheit und Medizin, Berlin, Germany; 4 Department of Psychiatry and Clinical Neuroscences CCM, Charité-Universitätsmedizin Berlin, Corporate Member of Freie Universität Berlin and Humboldt-Universität Zu Berlin, Berlin, Germany; 5 German Center for Mental Health (DZPG), partner site Berlin-Potsdam, Site Jena Magdeburg Halle, Magdeburg, Germany; 6 Department of Diagnostic and Interventional Radiology and Nuclear Medicine, University Medical Center Hamburg-Eppendorf, Hamburg, Germany; 7 Department of Psychiatry and Psychotherapy, Jena University Hospital, Jena, Germany; 8 Center for Intervention and Research on Adaptive and Maladaptive Brain Circuits, Underlying Mental Health (C-I-R-C), Site Jena Magdeburg Halle, Magdeburg, Germany; 9 Department of Psychiatry, Forensic Psychiatry, Ludwig Maximilians University Munich, Munich, Germany; 10 Department of Psychiatry, Psychotherapy and Preventive Medicine, Division of Forensic Psychiatry, LWL-University Hospital, Bochum, Germany

**Keywords:** Pedohebephilia, Viewing time, Incarceration, Child sexual offense, Viewing Time Index, DSM-5

## Abstract

With this study, we explored the complex interplay between sexual preference, behavior, and viewing time (VT) as an assessment tool for pedohebephilic interest by using a 2 × 2 factorial design. The design included two factors: sexual preference (pedohebephilic vs. teleiophilic) and history of child sexual abuse (with vs. without a prior child sexual offense [CSO]). A total of 320 men participated, including individuals with a sexual preference for children (*n* = 151) and those with a preference for adults (*n* = 169), encompassing those who had committed a CSO (*n* = 102) and those who had not (*n* = 218). By crossing these factors, the study investigated how preference and offense history interact to influence VT. Both ipsatized and raw VT data were analyzed. The clinical diagnosis of pedohebephilia and a prior CSO showed a significant association with the VT index. The study also revealed methodological insights, with raw VT data showing a primary effect of pedohebephilia and of CSO not visible in the ipsatized data. In a subsample of men with pedohebephilia, those who had not committed CSOs had longer absolute and relative VTs. In a subsample of men with teleiophilia, those who had committed CSO showed lower absolute and relative VTs for adults compared to healthy controls. They also showed higher relative VTs for Tanner stages 2–3. This study deepened our understanding of the complex interplay between sexual preference, behavior, and assessment methodologies, suggesting pathways for developing targeted interventions and preventive strategies for CSOs.

**Supplementary Information:**

The online version contains supplementary material available at 10.1007/s10508-025-03286-0.

## Introduction

### Pedohebephilia and Child Sexual Offense

Sexual interest in children is a significant risk factor for recidivism of child sexual offenses (CSO) (with a small effect of *d* = 0.32) (Eberhaut et al., 2023; Hanson & Morton-Bourgon, 2005). Still, sexual interest cannot be readily inferred from sexual behavior or vice versa. Research findings have repeatedly reflected that many men with pedophilia never abuse children (Beier et al., 2009; Dombert et al., 2016; Joyal & Carpentier, 2022) and studies of individuals convicted of a CSO report that up to over 50% of CSOs may be committed by men primarily attracted to adults (Schmidt et al., 2013; Welsch et al., 2021). Further complexity is added by the distinctions between pedophilia (attraction to pre-pubertal children) and hebephilia (attraction to early pubertal children), although recent academic discourse advocates for a broader classification of pedohebephilia (Stephens & Seto, 2016; Stephens et al., 2018), and differentiation between sexual interest in children, defined as any absolute sexual response to child stimuli, and a sexual preference for children, characterized by a relatively stronger sexual response to child stimuli compared to adult stimuli (Schmidt et al., 2014).

### Viewing Time as an Objective Measure of Sexual Interest

Traditional methods for measuring sexual interest are fraught with limitations, such as socially desirable responding and the potential for conscious manipulation in the case of self-report questionnaires or cost and intrusiveness in the case of penile plethysmography (Carvalho et al., 2020; Pohl, 2015; Wilson & Miner, 2016). Viewing time (VT) is a measure that potentially circumvents this problem by using imperceptibly measured reaction times: Participants are asked to rate the attractiveness of the individuals displayed in various sexual maturation stages. However, the actual time participants view the images is evaluated. The higher the sexual interest, the more features such as target gender, age, and attractiveness are considered in the assessment, prolonging the VT. Sexually unappealing stimuli can be excluded more quickly since the appraisal process is terminated as soon as one individual feature is considered unattractive (Pohl, 2015; Schmidt et al., 2017, 2021). Unfortunately, while this method is deliberate, it is susceptible to faking: Participants instructed to adopt the viewpoint of an individual with a differing sexual preference were able to achieve a VT score that aligned with the assumed sexual preference (Imhoff et al., 2012).

### Viewing Time in Relation to Other Measures of Sexual Interest

In one meta-analysis, VT presented as the most efficient indirect measure of pedophilic interest; it showed small-to-moderate convergent validity with self-reported data (average *r*_fixed-effect_ =.38, 95% CI [.29,.46]) and penile plethysmography (average *r*_fixed-effect_ =.25, 95% CI [.14,.35]) (Schmidt et al., 2017).

### Viewing Time and Sexual Offending

In the same meta-analysis, VT was significantly more accurate at discriminating people who had committed a CSO from healthy controls than from people who had sexually offended against adults (*p* < .01, as evidenced by nonoverlapping 95% confidence intervals). This suggests that offenses might influence VT (Schmidt et al., 2017). Gray et al. (2015) showed that VT of child-related stimuli was a significant predictor of sexual recidivism among men with a CSO history.

### Limitations of Viewing Time

Despite its utility, the field of VT measurement faces numerous challenges. Societal stigmatization and potential legal repercussions of associated pedohebephilic behaviors make accurate research in this field very complex, often leading to inconclusive findings. This, in addition to methodological limitations such as small sample sizes and a lack of control groups, have hindered a thorough understanding of how sexual behavior, particularly unreported or undetected offenses, might influence VT results. This is particularly evident in forensic settings where VT has predominantly been studied, but often without distinguishing the influences of pedophilia and CSOs by not specifying the proportion of participants who were pedohebephilic men or the proportion of non-admitters or controlling for undetected offenses (Jahnke, 2018; Schmidt et al., 2017).

### Viewing Time and Child Sexual Offense

One way to examine this potential dependence of VT on CSO is by comparing individuals with pedohebephilia who have committed a CSO with those who have not. Such an examination has been conducted in two online samples of self-identifying individuals with pedohebephilia (Jahnke et al., 2022). This study found no VT differences between individuals with pedohebephilia who self-reported previous CSO convictions, including child sexual abuse material (CSAM) offenses, and those who did not (first sample: T1–T5, *t*[146] = 1.15, *p* = .253, *d* = 0.29; second sample: *t*[487] = 0.02, *p* = .982, *d* = 0.00). However, the study only included a relatively small number of men with pedohebephilia who reported CSO convictions or CSAM (*n* = 30 in their first and *n* = 21 in their second sample), and offending status was determined based solely on self-reported convictions, making it impossible to deduce information about undetected offenses or to determine whether effects were a function of criminal sexual behavior or of being convicted for such acts. Incarceration rates were not reported, which is unfortunate, as many study participants in prior publications came from forensic settings and studies have shown that incarceration has a negative impact on cognitive functions (Umbach et al., 2018) and could therefore influence VT. Additionally, the pedohebephilic status was based on self-identification and not on clinical assessment, increasing the likelihood of men not reporting their pedohebephilic preference due to fear of stigmatization.

Therefore, Amelung et al. (2024) conducted a thorough examination of the potential influence of detected and undetected adult/child sexual behavior on VT in individuals with clinically ascertained pedohebephilia. Their study aimed to examine the relationship between prior sexual and non-sexual behaviors and VT in a sample of 282 self-referring, help-seeking men with and without pedohebephilia, with and without a history of prior CSOs or the use of CSAM, recruited outside a forensic context. Amelung et al. used equivalence intervals of -LL_95-CI_ < *ß* < 0 to test for equivalence of the response latencies in individuals with pedohebephilia with and without prior sexual offenses in multiple regression analyses. None of the estimators of the interaction estimates fell within the equivalence range. Thus, their analysis could not rule out a positive association between VT and each prior behavior, and even suggested a possible negative association when CSO and CSAM history were combined. The authors did not investigate teleiophilic men who had committed a CSO to determine whether they differed from healthy controls and could not possibly draw conclusions about the potential impact of CSOs on VT.

Pezzoli et al. (2022) took a different approach. In an anonymous online sample, fathers of girls were asked about attractiveness ratings, detected offenses, and the self-reported likelihood to make sexual contact with a child, while VT was measured unobtrusively. VT results revealed those with a self-reported likelihood to make sexual contact with children showed a similar VT behavior for all Tanner stages with no increase for adult stimuli. By contrast, those with no such self-reported likelihood showed an increase for adult stimuli. This result might reflect a relatively reduced sexual interest in adults in this group, a possibility that has received some empirical support (Knott et al., 2016; Schippers et al., 2023; Walter et al., 2007). Unlike VT studies which involved clinical or forensic samples, VT and attractiveness ratings failed to discriminate the nine participants with a detected sexual offending history (*AUC* =.51 [.34,.69], *p* = .900 and *AUC* =.50 [.36,.66], *p* = .980, respectively). The sample size of nine participants who had reported a history of sexual contact with a child was, of course, too small for solid conclusions.

### Study Rationale

The prior findings invite speculation whether the meta-analytically reported differences between men who have committed CSO and control groups (Schmidt et al., 2017) may be attributable to variables other than sexual interest, like factors that are associated with committing CSOs or having been arrested or convicted. To rule out such an alternative interpretation, a replication of VT differences between individuals with a sexual preference for children and those with a preference for adults, including those who have committed CSOs and those who have not, seems mandatory. With a sample of over 300 individuals from a clinical, a forensic, and a community background, we wanted to evaluate whether teleiophilic individuals who had committed CSOs showed changes in VT compared to healthy controls. Based on previous literature, we expect these individuals with teleiophilic offenders (C-PSO) to exhibit shorter VT for adult stimuli (T5) compared to teleiophilic non-offenders (HC). Second, we aimed to assess the VT difference between hands-on offenders and non-offenders in a pedohebephilic subsample. We did not dive into the VT differences between men with a pedohebephilic preference and men with a teleiophilic preference since they had been previously meta-analytically reported (Schmidt et al., 2017). The diagnoses of pedohebephilia were made in our study with the fourth edition of the *Diagnostic and Statistical Manual of Mental Disorders* (DSM-IV) criteria and were not, as often is the case, based on estimates or self-reports (for an overview, see also the meta-analysis by Pedneault et al., 2021).

To gain a better understanding of the complex interplay of VT, sexual preference, and behavior, we decided to report ipsatized and raw data for the specific Tanner stages. Ipsatized data are reported in the supplement. Additionally, we calculated a relative VT index because relative—rather than absolute—VT indices have shown greater validity, as demonstrated by Schmidt et al. (2017). Here, we expect pedohebephilic men to show positive average scores on the relative VT index (indicating longer RTs to child stimuli at T1–T3 than adult stimuli at T4-T5), while teleiophilic men to show negative scores (indicating longer RTs to adult stimuli than to child stimuli). By dividing the teleiophilic and the pedohebephilic groups into those who had been incarcerated and those who had not, we further explored the effects of incarceration in the supplement in order to discern whether the effects were a function of criminal sexual behavior or of being convicted for such acts.

## Method

### Participants

The data were gathered for a study named “Neuronal Mechanisms Underlying Pedophilia” (NeMUP) and are described in detail in Gerwinn et al. (2018). The participants were recruited via the official “NeMUP”-website, the CSO-prevention project “Don’t Offend” (www.dont-offend.org) for self-identified pedohebephilic people seeking help (Beier et al., 2009), various German internet forums for self-identified pedohebephilic people, the study centers’ websites, as well as correctional institutions at the five study sites in Germany (Berlin, Duisburg-Essen, Hannover, Kiel, Magdeburg) between January 2012 and January 2016. The healthy control group individuals were recruited via a neutral advertisement at each study site.

Exclusion criteria were neurological or acute psychiatric disorders like acute episodes of alcohol or drug abuse/dependence, depression, anxiety, intellectual disability, or psychotic disorders, assessed with a tailor made semi-structured interview in addition to the structured clinical interview for the DSM-IV-TR (Wittchen et al., 1997), and current medication related to sexual functioning or the diagnosis of pedohebephilia. All participants were 18–60 years old, male, and had refrained from using psychotropic medication for a minimum of 3 weeks prior to assessment.

All participants were financially compensated for their involvement in the study. Prior to their participation, they provided written, informed consent.

### Procedure

During their initial meeting, after thorough clinical evaluation, the participants were distributed into four groups: (1) pedohebephiles with a history of CSOs (P + CSO; *n* = 74), (2) pedohebephiles without a history of CSOs (P-CSO; *n* = 77), (3) teleiophilic participants with a history of CSO (CSO-P; *n* = 28), and (4) healthy controls (HC; *n* = 141). Sexual preference was determined by an assessment of the participants'sexual history, along with personal self-reported data derived from a tailored version of the Kinsey scale for sexual fantasy and behavior. Participants indicated their preferred age and sex for sexual partners by using pictures depicting Tanner stages 1–5 (Tanner, 1990). Pedohebephilia was diagnosed based on the tenth edition of the *International Classification of Diseases* (ICD-10) criteria for pedophilia (WHO, 2019) if participants reported sexually arousing fantasies and urges for children of pre-pubertal or early pubertal age (Tanner 1–3). Participants who indicated their preferred age for sexual partners for Tanner 4–5 were categorized as teleiophilic. Offender status was identified based on self-reports during a semi-structured interview specifically designed for this purpose. Participants were asked if they had committed a sex offense and, if yes, to provide details about the age, sex, and Tanner stage of their victim(s) as well as the type of sexual activity. We assumed these self-reports to be highly accurate, as anonymous participation was possible and medical doctors and licensed clinical psychotherapists in Germany are under strict therapeutic confidentiality and not obliged to report past sexual offenses unknown to authorities. We communicated this clearly to participants, making them less likely to withhold past offenses.

For participants who met the ICD-10 criteria for pedophilia and participants who did not meet these criteria but who had had a CSO history, additional data from previous and current treatments or forensic records were available to confirm self-assessments.

A participant was eligible for the P + CSO group if he acknowledged at least one sex offense involving touching or manipulating a child’s naked body under the age of 14 years or genitals, or penetration, for self-sexual stimulation or encouraging a child under the age of 14 to touch or manipulate the offender’s genitals or penetrate him. Pedohebephilic men without a history of hands-on offenses and/or who were solely consumers of CSAM or indicative material were assigned to the P-CSO group. Indicative materials, as defined in the COPINE scale, refer to non-erotic and non-sexualized pictures that show children in natural and unprovocative settings, like family albums or commercial sources. They differ from CSAM in the content of the images and the intent behind their creation and distribution. This categorization of the P-CSO group does not dismiss the illegality of CSAM in Germany (Taylor et al., 2001) but serves to distinguish between pedohebephilic men based on their engagement in hands-on CSOs. A participant was considered for the CSO-P group if he had committed a hands-on CSO without any clinical evidence of pedohebephilic preference. The control group included men without evidence of pedohebephilic preference or history of CSOs.

Additionally, we asked participants whether they had been incarcerated due to their CSO and/or CSAM. Incarceration was defined as deprivation of liberty, either through imprisonment in the regular prison system (German: JVA) or confinement in the forensic psychiatric system due to impaired criminal responsibility (German: Maßregelvollzug). This resulted in four groups who are presented in the supplement: (1) pedohebephilic men who had been in prison (P + I; *n* = 30), (2) pedohebephilic men who had not been in prison (P-I; *n* = 121), (3) non-pedohebephilic men who had been in prison (I-P; *n* = 21), and (4) HCs (*n* = 148).

### Measures

#### Sexual Variables

Participants were questioned about their history of consuming CSAM or indicative pictures.

#### Viewing Time

Sexual age preference is commonly defined via physical maturation and not actual age. Since physical maturation is subject to significant variability (across genders, individuals, and time periods) (Kowal et al., 2013; Krishna & Witchel, 2024), Tanner created a classification system for physical maturity stages: prepubescent (Tanner 1), early pubescent (Tanner 2), pubescent (Tanner 3), late pubescent (Tanner 4), or postpubescent (Tanner 5) (Tanner, 1990).

VT can be captured for each Tanner stage for both male and female images. This results, for example, in participants with interest in minors to look longest at images of male or female T1–T3 and shorter at images of T4–T5 males and females, whereas this effect is reversed in participants with interest in adults (Banse et al., 2010; Pohl, 2015).

We used a total of 80 images from the Not Real People Set (NRPS; Pacific Psychological Assessment Cooperation, 2004) in 10 different categories (each Tanner stage, T1–T5 for both sexes, eight images per category) as stimuli. The NRPS was created specifically for indirectly measuring sexual orientation and depicts computer-generated individuals in swimwear with a gray background. The experiment was programmed in Presentation© (https://www.neurobs.com/).

Participants were provided with a 4-point Likert scale to indicate how sexually arousing they found each image, analogous to Amelung et al.’s (2024) approach. The answers ranged from"not at all arousing"to"very arousing"and were given with the right hand using the keys"5"to"8"on a keyboard.

The images were presented for 5 s or until a response was provided—whichever came first. If there was no response within 5 s, the next image was presented and the run was scored with a reaction time of 5000 ms (in total, 140 trials with a reaction time [RT] > 5000 ms out of 25,680 presented trials for the whole sample, i.e., ~ 0.5%). The pictures were presented in a randomized order for every participant with a variable mean interstimulus interval of 1992 ms (range 1492–2492 ms). The randomized order for every participant was achieved by shuffling the images to present at the beginning of the experiment. Again, the participants were not informed that, instead of measuring their Likert scale score, the time between the presentation of the image to the entry of the response was recorded and analyzed.

### Data Preparation and Analysis

To account for sexual orientation, we based our calculations on the VT of the participants'preferred sex instead of the male and female images. To this end, we asked participants to indicate their sexual orientation. Following that question, we asked them to indicate a primary sexual orientation with the choices limited to heterosexual or homosexual.

The raw and ipsatized data were analyzed, and trials with an RT < 100 ms were discarded (41 trials out of 25,680 presented trials in total, representing ~ 0.16%). To achieve ipsatization for the analysis in the supplement, the data were *z*-transformed following the recommendation of Fisher (1915). Ipsatization allows one to compare results across different individuals. A downside of ipsatization is its potential to mask the low, specific sexual interest of one individual as being similar to the high, nonspecific sexual interest of another, despite the fact that these two profiles can have markedly different implications for treatment and assessment. In the past, both raw and ipsatized VT data have been published. Only a few researchers have reported both raw and ipsatized data. They have observed that correlations between VT and other methods of measuring pedophilic interest existed only (Mackaronis, 2014) or are greater (Pohl, 2015) when using raw data but not when using ipsatized data.

In addition, a VT index was calculated by subtracting the maximum RT from one of the adult categories (T4–T5) from the maximum RT from any of the child categories (T1–T3) of the preferred sex. Here, a positive VT-index pointed toward a pedohebephilic sexual interest, while negative values pointed toward a teleiophilic sexual interest.

For the analysis of the VT RTs, a 5 × 2 × 2 repeated-measure (RM) ANOVA, comprising the within-factor Tanner stage (T1–T5) and the between-subject factors pedohebephilia (P) (yes/no) and CSO (yes/no), was used. When sphericity was not given, results corrected via the Greenhouse–Geisser method are presented.

We opted for a 5 × 2 × 2 instead of a 5 × 4 ANOVA since the VT differences between men with pedohebephilic preferences and men with teleiophilic preferences had been previously meta-analytically reported (Schmidt et al., 2017), and we aimed to concentrate on the differences between the groups without or with CSOs. We therefore calculated post hoc *t* tests for each Tanner stage between P-CSO and P + CSO and between HCs and CSO-P when indicated.

We later replaced the between-subject factor CSO (yes/no) in the 5 × 2 × 2 RM-ANOVA by the factor incarceration (I) (yes/no) and present the results in the supplements.

## Results

### Descriptive Analyses

#### Group Characteristics

Age differed between offenders and non-offenders with offenders being older on average, *F*(1, 31) = 34.53, *p* < .001, partial *η*^2^ =.099, but not between pedohebephilic and teleiophilic men, *F*(1, 31) =.044, *p* = .843, partial *η*^2^ =.001. The interaction between P × CSO was nonsignificant, *F*(1, 31) = 0.58, *p* = .446, partial *η*^2^ =.001.

There was a high overlap of individuals who had been incarcerated, with 75% (21/28) in the CSO-P group and 38% in the P + CSO group (29/74). In the P-CSO group, one individual out of 76 had been incarcerated due to the selling of CSAM (1.3%). No individual had been incarcerated in the HC group, χ^2^(3) = 141.81, *p* < .001, Cramer’s *V* =.666.

Homosexual preference differed between the groups, with 46% in the P + CSO (34/74), 32% in the P-CSO (25/77), 16% in the CSO-P (4/28) and 27% in the HC (38/141) groups, χ^2^(3) = 12.37, *p* = .006, Cramer’s *V* =.197.

The two pedohebephilic groups reported a similar consumption of CSAM or indicative pictures and only a minority had never used indicative or explicit material (33% (25/75) in the P-CSO and 15% (11/71) in the P + CSO groups; 48% (12/25) in the CSO-P and 0.7% (1/141) in the HC group reported consumption of CSAM or indicative pictures). Data of three P + CSO, two P-CSO and one CSO-P respondents were missing, χ^2^(3) = 237.96, *p* < .001, Cramer’s *V* =.871 (Table 1).

**Table 1:** Characteristics of study groups

Variable		Group			
	P + CSO (*n* = 74)	P-CSO (*n* = 77)	CSO-P (*n* = 28)	HC (*n* = 141)	Statistic
	*M* (SD)	*M* (SD)	*M* (SD)	*M* (SD)	*p* = (P/CSO/PXCSO)
Age in years	41.0 (9.9)	34.6 (9.4)	42.3 (11.6)	33.8 (10.0)	*p* =.843/<.001/.446
Imprisonment (yes/no)	29/45	1/76	21/7	0/141	*p* < .001
*Sexual Variables*					
Primary orientation (hetero-/homosexual)	40/34	52/25	24/4	103/38	*p* = .006
Lifetime consumption of child abuse material (CSAM) or indicative pictures (yes/no)^a^	60/11	68/7	2/25	1/140	*p* < .001
Relative VRT index^b^	48.46 (448.4)	154.31 (486.2)	−510.78 (545.1)	−949.2 (651.7)	*p* < .001/.026/<.001

P + CSO, pedohebephilic men with a history of child sex offending; P-CSO, pedohebephilic men without a history of hands-on child sex offending; CSO-P, non-pedohebephilic men with a history of child sex offending, HC, healthy controls

^a^Missing data: P + CSO n = 3; P-CSO n = 2; CSO-P n = 1

^b^Relative VT index is obtained by subtracting the maximum reaction time of the preferred adult category (Tanner 4–5) from the maximum reaction time of the preferred child category (Tanner 1–3) for the sexual orientation-matched VT

### Attractiveness Rating

The 5 × 2 × 2 ANOVA on attractiveness rating comprising the within-factor Tanner stage (T1–T5) and the between-subject factors pedohebephilia (P) (yes/no) and CSO (yes/no) showed a main effect of Tanner, *F*(1.58, 312.55) = 21.42, *p* < .001, partial *η*^2^ =.063, and P, *F*(1, 316) = 96.94, *p* < .001, partial *η*^2^ =.235, but not CSO, *F*(1, 316) = 0.36, *p* = .545, partial *η*^2^ =.001. In addition, the analysis showed an interaction of Tanner × P, *F*(1.58, 312.55) = 99.91, *p* > .001, partial *η*^2^ =.240, P × CSO, *F*(1, 316) = 7.74, *p* = .006, partial *η*^2^ =.024, but no interaction between Tanner × CSO, *F*(1.58, 312.55) = 2.73, *p* = .079, partial *η*^2^ =.009, nor a CSO × P × Tanner interaction, *F*(1.58, 312.55) = 1.79, *p* = .187, partial *η*^2^ =.005. To get a better impression of the differences between pedohebephilic and non-pedohebephilic participants, 2 × 5 ANOVAs for the pedohebephilic and non-pedohebephilic subgroups were conducted.

The attractiveness ratings for the non-pedohebephilic subgroups showed a main effect of Tanner, *F*(1.53, 256.04) = 130.98, *p* < .001, partial *η*^2^ =.44, CSO, *F*(1, 167) = 5.76, *p* = .017, partial *η*^2^ =.033, as well as an interaction, *F*(1.53, 256.04) = 5.69, *p* = .008, partial *η*^2^ =.033. A post hoc *t* test showed that CSO-Ps rated pictures of their preferred gender toward T3 as more attractive than HCs did. The attractiveness ratings for the pedophilic subgroups showed a main effect of Tanner, *F*(1.55, 231.51) = 21.22, *p* < .001, partial *η*^2^ =.125, but no interaction, *F*(1.55, 231.51) = 0.17, *p* = .784, partial *η*^2^ =.001, nor a main effect of CSO, *F*(1, 149) = 2.54, *p* = .113, partial *η*^2^ =.017. We observed no difference in ratings between the P + CSO and P-CSO groups (Table 2).

**Table 2:** Mean attractiveness rating for the preferred gender for the five Tanner stages of the four groups (P = Pedohebephilia, CSO = child sexual offense, HC = Healthy controls) on a 4-point Likert scale

Tanner	Group		Statistic	Cohen's *D*
	P + CSO (*n* = 74)	P–CSO (*n* = 77)		
	Mean (SD)	Mean (SD)		
T1	2.2 (0.9)	2.3 (0.8)	*p* = .525	0.10
T2	2.3 (0.9)	2.5 (0.8)	*p* = .328	0.16
T3	2.3 (0.8)	2.5 (0.6)	*p* = .213	0.20
T4	2.2 (0.7)	2.4 (0.7)	*p* = .110	0.26
T5	1.8 (0.7)	2.0 (0.9)	*p* = .147	0.24

	CSO-P (*n* = 28)	HC (*n* = 141)		
T1	1.3 (0.8)	1.1 (0.4)	*p* = .096	0.56
T2	1.4 (0.9)	1.1 (0.4)	*p* = .053	0.67
T3	1.5 (0.9)	1.1 (0.4)	***p* = .029**	0.74
T4	1.9 (0.8)	1.7 (0.6)	*p* = .102	0.43
T5	2.2 (0.8)	2.3 (0.7)	*p* = .495	0.14

Bold font indicates that post-hoc *t*-test reached statistical significance (*p* < 0.05, Bonferroni corrected)

### Relative Viewing Time Index

**Fig. 1:**
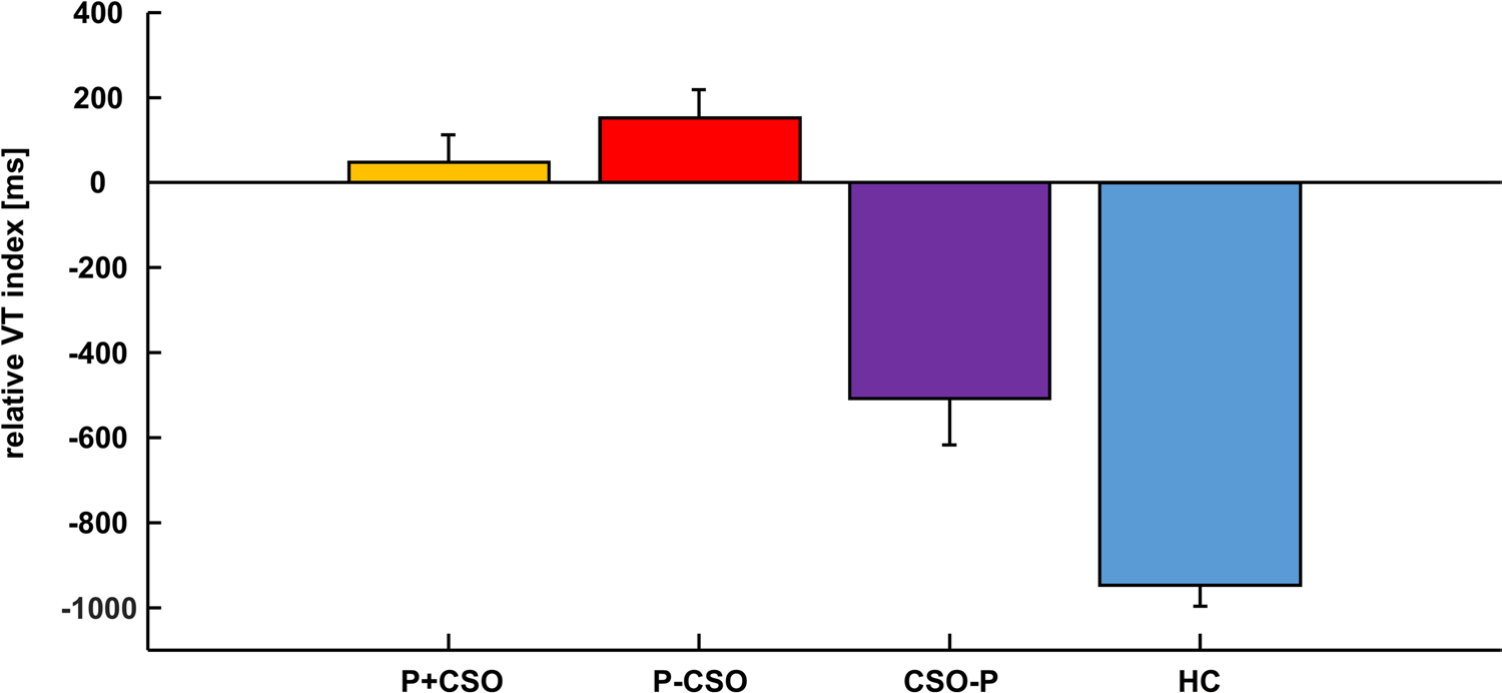
Relative Viewing Time index (ms) for the preferred gender of the four groups (P = Pedohebephilia, CSO = Child sexual offense, HC = Healthy controls)

Pedohebephilic men showed positive average scores on the relative VT index (indicating longer RTs to child stimuli at T1–T3 than adult stimuli at T4-T5), while teleiophilic men had negative scores (indicating longer RTs to adult stimuli than to child stimuli). The analysis of the VT index detected a significant main effect of P, *F*(1, 316) = 125.89, *p* < .001, partial *η*^2^ =.285, of CSO, *F*(1, 316) = 5.03, *p* = .026, partial *η*^2^ =.016, and an interaction between P × CSO, *F*(1, 316) = 13.48, *p* < .001, partial *η*^2^ =.041. A post hoc *t* test showed that HCs had the lowest indices (*p* < .001 for all comparisons), followed by CSO-Ps (*p*  < .001 for all comparisons), while the P + CSO and P-CSO did not differ (*p* = .249) but were higher compared to the non-pedohebephilic groups (*p* < .001) (Table 1; Fig. 1).

### Mean Raw Viewing Time Data

We calculated a 5 × 2 × 2 rm-ANOVA that comprised the within-factor Tanner stage (T1–T5) and the between-subject factors pedohebephilia (P) (yes/no) and CSO (yes/no).

The analysis detected a significant main effect of P, *F*(1, 316) = 24.27, *p* < .001, partial *η*^2^ = 0.071, of T, *F*(2.20, 695.40) = 37.04, *p* < .001, partial *η*^2^ = 0.105, and of CSO, *F*(1, 316) = 4.92, *p* = .027, partial *η*^2^ = 0.015.

**Fig. 2:**
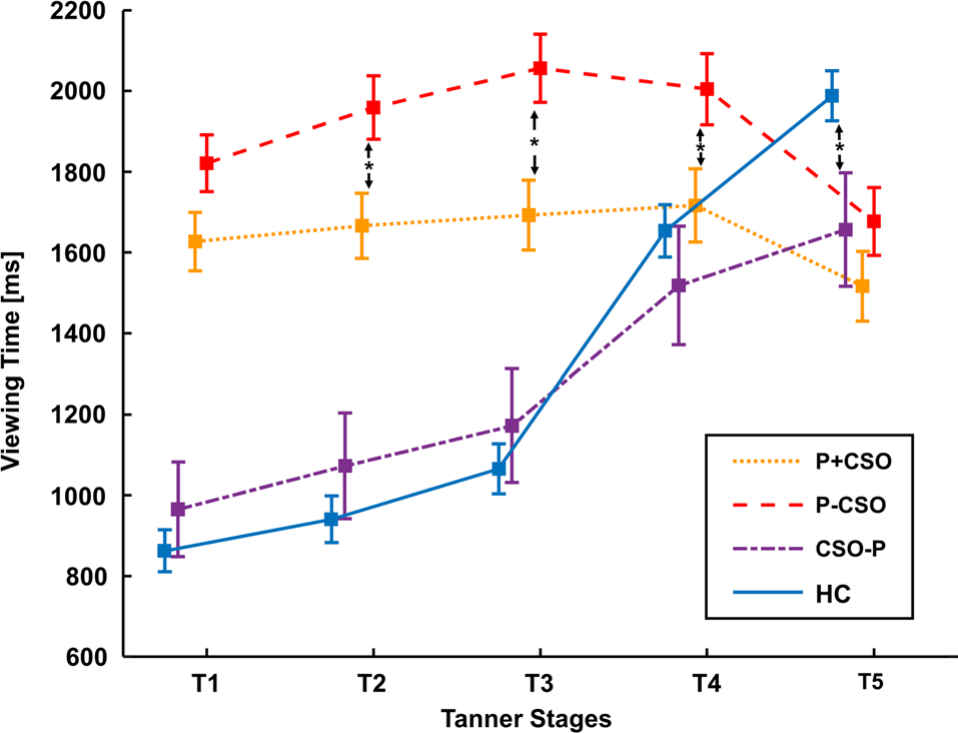
Mean raw Viewing Time data (ms) for the preferred gender for the five Tanner stages of the four groups (P = Pedohebephilia, CSO = Child sexual offense, HC = Healthy controls). * indicates significant post-hoc differences between groups (*p* < 0.05, Bonferroni corrected)

Additionally, there were significant interactions between T × P, *F*(2.20, 812.73) = 60.57, *p* < .001, partial *η*^2^ = 0.161, and T × P × CSO, *F*(2.20, 695.40) = 6.55, *p* = .001, partial *η*^2^ = 0.020, but no significant interactions between T × CSO, *F*(2.20, 695.40) = 2.73, *p* = .060, partial *η*^2^ = 0.009, and P × CSO, *F*(1, 316) = 1.25, *p* = .264, partial *η*^2^ = 0.004, were observed. As shown in Table 3, post hoc *t* tests revealed significant differences in the raw data for T1–T4 between P-CSO and P + CSO and for T5 between CSO-P and HC individuals (Fig. 2).

**Table 3:** Mean raw viewing time data (ms) for the preferred gender for the five Tanner stages of the four groups (P = Pedohebephilia, CSO = Child sexual offense, HC = Healthy controls)

	Group		Statistic	Cohen's *d*
	P + CSO (*n* = 74)	P–CSO (*n* = 77)		
	Mean (SD) (ms)	Mean (SD) (ms)		
T1	1636.3 (788.8)	1883.7 (839.6)	***p* = .016**	0.30
T2	1682.5 (847.8)	2052.8 (1015.5)	***p* = .002**	0.40
T3	1729.3 (825.1)	2125.9 (1073.8)	***p* = .002**	0.41
T4	1722.5 (760.9)	2070.1 (106.3)	***p* = .022**	0.38
T5	1545.3 (660.1)	1707.0 (903.8)	*p* = .357	0.20

	CSO-P (*n* = 28)	HC (*n* = 141)		
	Mean (SD) (ms)	Mean (SD) (ms)		
T1	991.0 (433.0)	882.8 (386.4)	*p* = .406	0.27
T2	1065.3 (472.2)	978.4 (489.3)	*p* = .568	0.18
T3	1178.0 (618.5)	1097.1 (540.7)	*p* = .613	0.15
T4	1595.1 (795.7)	1824.7 (947.3)	*p* = .231	0.24
T5	1748.9 (798.6)	2297.9 (1349.3)	***p* = .014**	0.43

Bold font indicates that post-hoc *t*-test reached statistical significance (*p* < 0.05, Bonferroni corrected)

## Discussion

### Viewing Time Index

We analyzed raw RTs of a VT task and their dependence on pedohebephilia and CSO (see the supplement for results on ipsatized VT and incarceration) in a large sample from clinical, forensic, and community backgrounds. Replicating prior findings, we found positive average scores on the VT index for pedohebephilic men, while teleiophilic men achieved negative scores. This is in accordance with numerous studies (Amelung et al., 2024; Eberhaut et al., 2020; Schmidt & Perkins, 2020; Schmidt et al., 2017).

The analysis of the VT index detected an additional effect of CSO and an interaction between P × CSO. Here, HCs achieved the lowest values, CSO-P individuals were somewhere in between (but also negative) while the pedophilic groups showed similar positive values. This could indicate that CSO-P demonstrated a somewhat flatter/more unspecific viewing behavior (shorter VTs for T5 and longer VTs for T2–T3) compared to HCs, suggesting a broader-tuned sexual preference.

### Attractiveness Rating

Controversially, teleiophilic offenders rated stimuli as more attractive compared to HCs in T3, although there was no significant difference in rated stimuli in T5. It remains unclear whether an unconscious lower interest in adults might contribute to CSOs.

### Post Hoc *t* Tests of Tanner Stages

To better understand the interactions, we looked into the post hoc *t* tests of Tanner stages. In individuals without pedohebephilia, only the ipsatized VTs of T2 and T3 but not the absolute RTs differed between the HCs and non-pedohebephilic offenders (CSO-P), with offenders showing longer ipsatized VTs than non-offenders. Furthermore, both absolute and relative VTs toward T5 were lower in offenders. Perhaps this represents a lower sexual interest in T5 in CSO-Ps. Our data therefore confirm our hypothesis of different VTs in these groups and align with meta-analytic data that indicate that people who have committed CSOs score lower on sexual interest in adults than comparison groups (Schippers et al., 2023).

Concerning the ipsatized data, differences between the teleiophilic groups appeared in T2 and T3, with offenders showing longer VTs than HCs. This longer VT could be interpreted as an indicator of hebephilic interest in the teleiophilic offender group—something repeatedly described in other papers (Pedneault et al., 2021). Concentrating on a higher interest in T2–T3 and a lower interest in adults could become a promising intervention for the prevention of CSOs by teleiophilic men.

Pedohebephilic individuals with a history of CSO (P + CSO) showed shorter absolute RTs in age categories T1–T4 compared to non-offending individuals with pedohebephilia (P-CSO). These shorter relative response latencies in offending pedohebephiles were also visible in ipsatized RTs in T1–T3 but not in T4 or T5.

The first explanation of these data is based on the framework proposed by Schmidt et al. (2017). The VT effect might arise from the distinction between the dismissal of clearly irrelevant stimuli and the meticulous examination of potentially relevant sexual stimuli. The P-CSO group might be particularly proactive in seeking help and concerned about their potential pedohebephilic tendencies compared to the P + CSO group. Those troubled by this concern may show increased scrutiny in evaluating the arousal potential of stimuli related to children.

Second, VT is an indicator of heightened sexual interest, its original intended purpose by (Rosenzweig, 1942), when he used it to discern individuals with schizophrenia with “high” and “low” frequency sexual behavior by recording response latencies while watching sexually salient visual stimuli. Indeed, we already know from the Gerwinn et al. (2018) study that the P-CSO group was characterized by a higher total number of total sexual outlet (i.e., orgasms per week), sexual excitation score, and rate of sexual sadism than their offending counterparts. Also, research by Amelung et al. (2024) showed a correlation between the VT index and hypersexuality (rho = 0.26, *p* < 0.05) on the STABLE-2007, a scale for structured clinical assessment of dynamic risk factors of sexual offending in adult male sexual offenders (Hanson & Hanson, 2007). In addition, studies by Seto et al. (2006) and Webb et al. (2007) revealed that individuals convicted of CSAM offenses exhibit more significant problems with sexual self-regulation. They displayed greater sexual arousal toward children and had more frequent sexual fantasies about children than those convicted of CSOs. However, the Seto and Webb studies did not specify whether and how many individuals with pedophilic inclinations were included in the CSO group and the CSAM offender group. This somewhat paradoxical observation—that lower sexual interest in child sexual stimuli may be associated with CSOs—deserves further examination.

Third, it may be postulated that individuals without hands-on offenses (P-CSO), accustomed to indulging their preferences through CSAM, exhibit longer VTs when evaluating stimuli due to a reversion to their habitual patterns of behavior. This hypothesis suggests that the extended VTs observed in the P-CSO group reflect a deeper engagement with the stimuli, possibly because these individuals have developed specific patterns of sexual arousal and gratification through non-direct means like CSAM. This pattern of behavior could serve as a coping mechanism or alternative to hands-on offenses, allowing some individuals to navigate their preferences within a safer, albeit legally and morally objectionable, framework. The implication here is twofold: First, that the longer VT signifies an entrenched behavioral pattern among P-CSO individuals, as they interact with stimuli reminiscent of their usual CSAM consumption habits; and second, that this engagement pattern may act as a psychological barrier or substitute for actual offending behavior. The longer VTs in P-CSOs might derive from this complex interplay between awareness, concern, and response pattern.

### Study Limitations and Outlook

When interpreting our results, several limitations should be considered regarding the included participants, the methodology, and selection of measures. For instance, pedohebephilia group participants might have been more burdened pedohebephilic men compared to a population-based or other non-clinical control samples. They were recruited via clinical and forensic environments and forums which addressed sexual interest in children, where individuals may seek support due to increased distress levels (Jahnke et al., 2015).

Moreover, we were not always able to verify abstinence from CSOs for the HC and P-CSO groups. However, we assumed the reports to be highly accurate, as we communicated to participants that in Germany, medical doctors and licensed clinical psychologists are bound by medical confidentiality and are not obligated to report past CSO cases unknown to authorities. If any, we expect only a small proportion of participants in the P-CSO group to have falsely denied CSOs.

Another important study limitation relates to the inherent heterogeneity and potential confounds associated with using offending status as a grouping variable. While our rationale was to contrast individuals with pedohebephilic interests who had (P + CSO) or had not (P-CSO) committed CSO, this classification inevitably aggregates individuals from distinct subpopulations (e.g., convicted offenders, self-referred individuals), whose psychological profiles may differ substantially. Furthermore, although most pedohebephilic individuals reported current or past use of CSAM material, only a single participant had a conviction for CSAM-only offenses. Additional analyses showed no significant main or interaction effects of CSAM use on viewing time data, suggesting limited added value for further subgrouping in this already complex design. However, we acknowledge that CSAM offending may reflect higher specificity of sexual interest and risk, as highlighted in prior meta-analyses (e.g., Babchishin et al., 2015; Seto et al., 2006), and that this nuance cannot be fully captured in our analyses.

We also recognize that several third variables (e.g., intelligence, sexual excitation/inhibition, or hypersexuality) may influence both viewing time and offending risk. While we did include self-reported sexual excitation and inhibition tendencies (SES/SIS) and masturbation frequency and assessed cognitive functioning with a short version of the German version of the third edition of the Wechsler Adult Intelligence Scale (Molz et al., 2010), these variables were not the focus of the current analyses.

Finally, we had partial information on victim characteristics, and exploratory analyses supported previous findings suggesting systematic differences between groups. Specifically, 82% of the CSO-P group had female victims, whereas this was true for only 48% of the P + CSO group, *χ*^2^(1) = 9.3, *p* = 0.002. Conversely, 58% of the P + CSO group had male victims compared to 35% of the CSO–P group, *χ*^2^(1) = 4.1, *p* = 0.043. A subset of participants in both groups reported victims of both genders (eight in the P + CSO group and five in the CSO-P group), so the percentages did not sum to 100%. These differences suggest that individuals with pedohebephilic interest who had committed contact offenses (P + CSO) were more likely to have male and possibly extrafamilial victims, which may reflect stronger or more specific sexual interests. There was a high overlap of individuals who had been incarcerated in the CSO-P and the P + CSO groups. In the P-CSO group, one individual had been incarcerated from selling CSAM. The discriminatory power of CSO could be affected by this, and perhaps the effects were mainly driven by incarceration, necessitating further research even if it is difficult to assess these variables separately. The controlled and restrictive environment of a prison and the high levels of stress and mental health issues might affect how inmates respond to sexual stimuli, potentially altering their VTs compared to a non-incarcerated population. While such differences support the relevance of distinguishing groups based on sexual interest and offense history, they also highlight the presence of potentially meaningful third variables that could not be fully controlled for in the current design. These findings likely reflect the joint influence of selection processes, individual traits, and offense-related dynamics. Future researchers might incorporate more detailed typologies and consider these characteristics more systematically.

Two other limitations stem from the original design as a multi-site research project that included a comprehensive test battery as well as magnetic resonance imaging (MRI) examinations: Since this study was initially designed to be conducted within an MRI setting, where we planned to use a restricted four-button response system due to the constrained environment, accordingly, we shortened the trial lengths to accommodate the high costs associated with MRI time. However, changes in the availability of MRI scanning time led to modifications in our experimental setup, but without a corresponding adaptation of the experimental paradigm. Despite these changes, the use of a four-button system probably had a minimal impact on the RT measurements, which were the primary focus of our analysis. Furthermore, since all participants faced the same response constraints, the uniformity across groups ensured comparability in our analysis of group differences. However, these constraints might limit the generalizability of our findings to other VT experiments that utilize different setups or more extensive response options.

Future research might dissect this and new datasets between hebephilic, pedophilic, and teleiophilic individuals. Although the contemporary research often combines the first two groups due to their apparent similarities, such future studies could thereby uncover potential differences that might deepen our understanding of these preferences and potential unique characteristics. Conducting the same method online like in Pezzoli et al. (2022) and Jahnke et al. (2022) could provide further insights because community-based participants display less distress than samples with a clinical or forensic background. The two pedohebephilic groups reported similar consumption of CSAM or indicative pictures and only a minority had never used indicative or explicit material (Table 1). It would be interesting to understand more about the effect explicit or indicative material consumption has on VT. It is important to further explore whether extended VTs are indicators of heightened sexual interest and/or the complex interplay between awareness, concern, and response pattern and to what extent they are inherent to the different groups and to CSOs or incarceration. Further investigation will hopefully help in developing targeted interventions and preventive strategies in the context of CSOs.

### Conclusion

Considering the complex interplay between sexual preference, behavior, and VT as an assessment tool sheds light on the nuanced understanding required to address CSOs. Our findings underscore the intricacy of discerning sexual interest from behavior, highlighting that not all individuals with pedohebephilic preferences engage in CSOs and that CSOs are committed by individuals across a spectrum of sexual preferences. Both the clinical diagnosis of pedohebephilia and prior CSOs showed significant associations with the VT index. The differentiation between the raw and ipsatized VT data offered valuable methodological insights, revealing that one’s analytical approach can significantly impact the interpretation data, with raw data showing a main effect of pedohebephilia and of CSO, while the ipsatized data did not.

Moreover, our investigation of Tanner stages has provided critical insights into the sexual attraction profiles of pedohebephilic and teleiophilic men, with significant differences observed across groups and stages, again underscoring the importance of nuanced assessment methods.

Granted, the limitations of this study, such as the potential biases introduced by the recruitment of participants from clinical and forensic settings and the high overlap between individuals who had committed CSOs and who had been imprisoned for this, highlight the need for further research. Future investigators should recruit broader, more diverse populations and examine the effects of incarceration on VTs. Additionally, exploring the impact of CSAM or indicative material on VTs could offer deeper insights.

Ultimately, this study supports ongoing efforts to disentangle the complex web of factors surrounding CSOs and pedohebephilic interest. By refining our understanding of these dynamics, the field can better address the challenges associated with preventing CSOs and supporting individuals with pedohebephilic preferences in non-offending pathways.

## Supplementary Information

Below is the link to the electronic supplementary material.

Supplementary file1 (DOCX 150 KB)

Supplementary file2 (DOCX 15 KB)

## Data Availability

The datasets analyzed are available from the corresponding author on reasonable request.
