# Supplementary material for: Viewing Time Behavior in a Diverse Sample of 320 Pedohebephilic and Teleiophilic Men Who Have Committed Child Sexual Offense and Who Have Not
Source: Arch Sex Behav. 2025 Dec 26;55(1):165–77. doi: 10.1007/s10508-025-03286-0 (PMC12917018; doi:10.1007/s10508-025-03286-0)
Supplement: Supplementary file 1 — Supplementary file1 (DOCX 150 KB) [file 10508_2025_3286_MOESM1_ESM.docx]

**Ipsatized VT data ANOVA comprising the within-factor Tanner stage (T1-T5), and the between-subject factors pedohebephilia (P) and CSO**

For the ipsatized data, the analysis showed no significant effect of P (*F*(1, 316) = 0.300, *p* = .585, partial η² = 0.001) or of CSO (*F*(1, 316) = 1.719, *p* =.191, partial η² = 0.005) but of T (*F*(2.975, 939.944) = 93.003, *p* < .001, partial η² = 0.227) on VT.

Additionally, there were significant interactions between T X P (*F*(2.975, 939.944) = 131.396, *p* < .001, partial η² = 0.294), T X P X CSO (*F*(2.975, 939.944) = 9.089, *p* < .001, partial η² = 0.028) and P X CSO (*F*(1,316) = 4.106, *p* = .044, partial η² = 0.013), but not between T X CSO (*F*(2.975, 939.944) = 0.593, *p* = .618, partial η² = 0.002).

In the ipsatized data, post hoc test showed significant differences in T2 and T3 between the HC and CSO-P and the P-CSO and P+CSO groups making the differences between participants who committed CSO and those who did not visible in T2 and T3. Additionally, in T1, a significant difference was found between the P-CSO and P+CSO groups and in T5 between HC and CSO-P (Supplement Table 3 and supplement Figure 1).

**Supplement Table 1:**

*Mean ipsatized VT data for the preferred gender for the 5 Tanner stages of the 4 groups (P=Pedohebephilia, CSO=Child sexual offense, HC=Healthy controls)*

|  | Group | |  |  |
| --- | --- | --- | --- | --- |
|  | **P+CSO (n = 74)** | **P–CSO (n = 77)** |  |  |
|  | Mean (*SD*) | Mean (*SD*) | Statistic | Cohens *D* |
| T1 | 0.248 (0.46) | 0.388 (0.43) | ***p* = .018** | 0.32 |
| T2 | 0.277 (0.53) | 0.455 (0.4) | ***p* = .005** | 0.38 |
| T3 | 0.352 (0.47) | 0.574 (0.5) | ***p* = .001** | 0.46 |
| T4 | 0.412 (0.51) | 0.484 (0.42) | *p* = .371 | 0.16 |
| T5 | 0.218 (0.69) | 0.100 (0.51) | *p* = .216 | 0.20 |
|  | **CSO-P (n = 28)** | **HC (n = 141)** |  |  |
|  | Mean (SD) | Mean (SD) |  |  |
| T1 | -0.170 (0.33) | -0.289 (0.25) | *p* = .113 | 0.44 |
| T2 | -0.019 (0.3) | -0.198 (0.3) | ***p* = .026** | 0.67 |
| T3 | 0.155 (0.52) | -0.039 (0.32) | ***p* = .029** | 0.53 |
| T4 | 0.772 (0.62) | 0.825 (0.49) | *p* = .607 | 0.10 |
| T5 | 0.988 (0.64) | 1.320 (0.55) | ***p* = .006** | 0.59 |


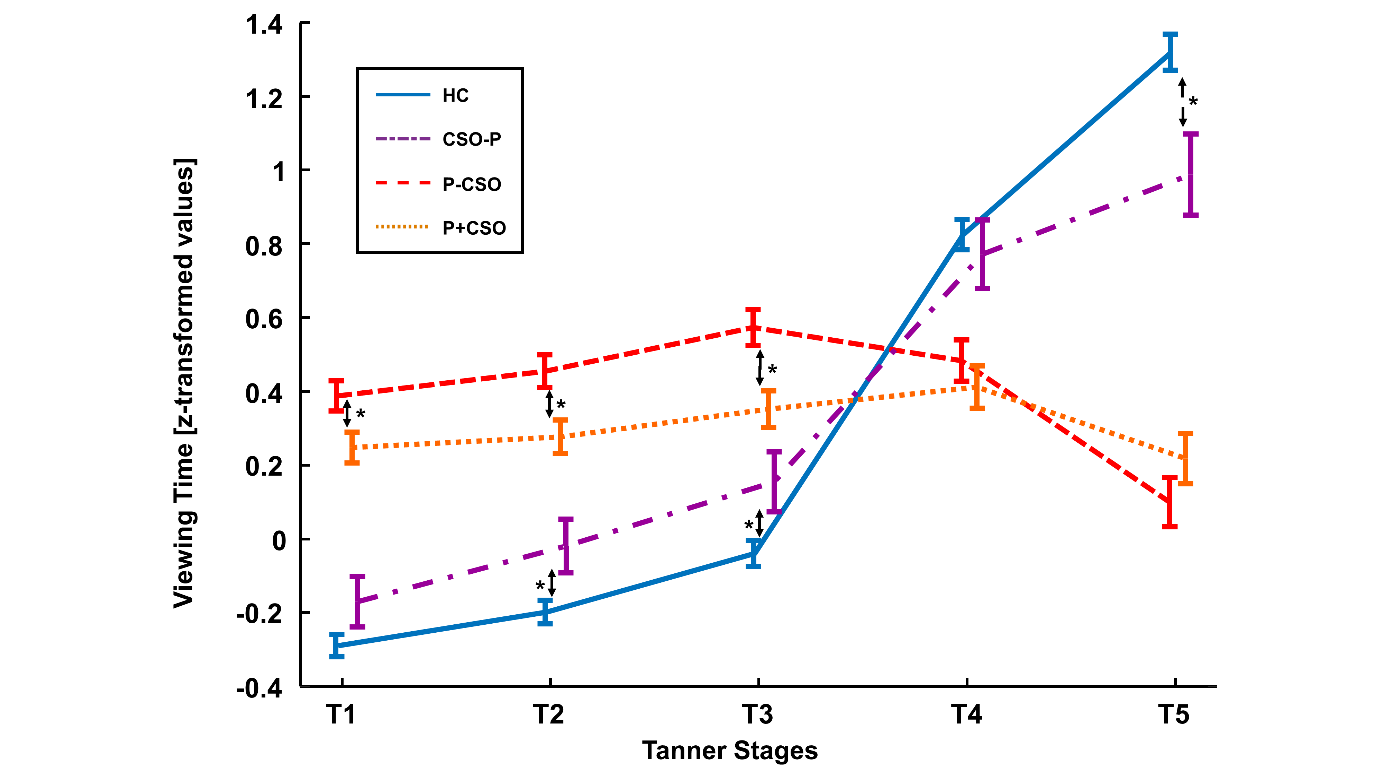


**Supplement Fig. 1:** **Mean ipsatized VT data for the preferred gender for the 5 Tanner stages of the 4 groups (P=Pedohebephilia, CSO=Child sexual offense, HC=Healthy controls)**

* indicates *p* < .05

**Mean raw VT data ANOVA comprising the within-factor Tanner stage (T1-T5), and the between-subject factors pedohebephilia (P) and incarceration (I)**

In the 5X2X2 rm-ANOVA by the factor incarceration (I) (yes/no), for the raw data, a considerable main effect for T (*F*(2.201, 695.421) = 25.290, *p* < .001, partial η² = 0.074), P (*F*(1, 315) = 11.189, *p* = .001, partial η² = 0.034) and I (*F*(1, 315) = 9.120, *p* = .003, partial η² = 0.028) was noted. Interactions between T × P (*F*(2.201, 695.421) = 37.310, *p* < .001, partial η² = 0.106), T × I (*F*(2.201, 695.421) = 2.613, *p* = .069, partial η² = 0.008), and T × I × P (*F*(2.201, 695.421) = 5.278, *p* = .004, partial η² = 0.016) were significant. The interaction between I × P (*F*(1, 315) = 1.416, *p* = .235, partial η² = 0.004) did not reach significance.

In the post hoc tests the same differences between groups as in the ANOVA by the factor CSO were significant (see Supplements Table 1).

**Supplements Table 2:**

**Mean raw VT data (ms) for the preferred gender for the 5 Tanner stages of the 4 groups (P=Pedohebephilia, I=Incarceration, HC=Healthy controls)**

|  | **Group** |  |  |  |
| --- | --- | --- | --- | --- |
|  | **P+I (n = 30)** | **P–I (n = 121)** |  |  |
|  | Mean (*SD*) (ms) | Mean (*SD*) (ms) | Statistic | Cohens *D* |
| T1 | 1446.5 (715.1) | 1840.8 (830.3) | ***p* = .002** | **0.49** |
| T2 | 1434.5 (851.8) | 1979.6 (947.9) | ***p* = .000** | **0.59** |
| T3 | 1512.1 (930.6) | 2035.5 (964.2) | ***p* = .001** | **0.55** |
| T4 | 1541.5 (815.0) | 1988.6 (949.6) | ***p* = .018** | **0.48** |
| T5 | 1357.9 (588.8) | 1694.6 (827.1) | *p* = .124 | 0.43 |
|  | **I-P (n = 21)** | **HC (n = 148)** |  |  |
|  | Mean (*SD*) (ms) | Mean (*SD*) (ms) |  |  |
| T1 | 961.3 (454.4) | 892.1 (387.0) | *p* = .636 | 0.18 |
| T2 | 995.7 (438.8) | 992.4 (493.9) | *p* = .985 | 0.01 |
| T3 | 1089.2 (540.4) | 1113.5 (556.7) | *p* = .893 | 0.05 |
| T4 | 1494.0 (803.9) | 1828.2 (936.7) | *p* = .122 | 0.36 |
| T5 | 1602.0 (731.7) | 2292.8 (1329.1) | ***p* = .006** | **0.54** |

**Ipsatized VT data ANOVA comprising the within-factor Tanner stage (T1-T5), and the between-subject factors pedohebephilia (P) and incarceration (I)**

In the 5X2X2 rm-ANOVA by the factor incarceration (I) (yes/no), for the ipsatized data, the analysis detected no significant main effect of P (*F*(1, 316) = 1.186, *p* = .277, partial η² = 0.004). However, there was a significant main effect of I (*F*(1, 316) = 7.638, *p* = .006, partial η² = 0.024) and of T (*F*(2.963, 936.202) = 68.883, *p* < .001, partial η² = 0.179). Additionally, there were significant interactions of T × P (*F*(2.963, 936.202) = 78.812, *p* < .001, partial η² = 0.200), I × P (*F*(1, 315) = 8.112, *p* = .005, partial η² = 0.025), and T × P × I (*F*(2.963, 936.202) = 8.244, *p* < .001, partial η² = 0.025), but not between T × I (*F*(2.963, 936.202) = 1.165, *p* = .322, partial η² = 0.004). Differing from the ANOVA by the factor CSO, the effect of incarceration was visible in both the ipsatized and the raw data.

In the post hoc tests the same differences between groups as in the ANOVA by the factor CSO were significant (Supplements Table 2) but the difference between the I-P and the HC group missed significance in T3 with *p*=.051. Therefore the differences between participants who were incarcerated and those who did not were only visible in T2 and not between T2 and T3 as seen in the ANOVA by the factor CSO.

**Supplements Table 3:**

**Mean ipsatized VT data for the preferred gender for the 5 Tanner stages of the 4 groups (P=Pedohebephilia, I=Incarceration, HC=Healthy controls)**

|  | **Group** |  |  |  |
| --- | --- | --- | --- | --- |
|  | **P+I (n = 30)** | **P–I (n = 121)** |  |  |
|  | Mean (*SD*) | Mean (*SD*) | Statistic | Cohens *D* |
| T1 | 0.183 (0.41) | 0.353 (0.45) | ***p* = .022** | **0.38** |
| T2 | 0.069 (0.50) | 0.442 (0.44) | ***p* < .001** | **0.83** |
| T3 | 0.236 (0.54) | 0.522 (0.47) | ***p* = .001** | **0.59** |
| T4 | 0.321 (0.50) | 0.480 (0.45) | *p* = .112 | 0.35 |
| T5 | 0.164 (0.77) | 0.156 (0.56) | *p* = .945 | 0.01 |
|  | **I-P (n = 21)** | **HC (n = 148)** |  |  |
|  | Mean (SD) | Mean (SD) |  |  |
| T1 | -0.140 (0.36) | -0.287 (0.25) | *p* = .080 | 0.55 |
| T2 | -0.008 (0.30) | -0.191 (0.30) | ***p* = .038** | **0.61** |
| T3 | 0.164 (0.46) | -0.031 (0.35) | *p* = .051 | 0.53 |
| T4 | 0.752 (0.68) | 0.825 (0.49) | *p* = .521 | 0.14 |
| T5 | 0.898 (0.66) | 1.317 (0.55) | ***p* = .002** | **0.74** |
