# Supplementary material for: Viewing Time Behavior in a Diverse Sample of 320 Pedohebephilic and Teleiophilic Men Who Have Committed Child Sexual Offense and Who Have Not
Source: Arch Sex Behav. 2025 Dec 26;55(1):165–77. doi: 10.1007/s10508-025-03286-0 (PMC12917018; doi:10.1007/s10508-025-03286-0)
Supplement: Supplementary file 2 — Supplementary file2 (DOCX 15 KB) [file 10508_2025_3286_MOESM2_ESM.docx]

**Appendix A. Effect sizes of the general study group characteristics**

| Variable | Partial eta squared (*partial η^2^*) |
| --- | --- |
| Age in years (P/CSO/PXCSO) | (.001/.099/.001) |
| Imprisonment (yes/no) | (.066/.439/.075) |
| *Sexual Variables* |  |
| Primary orientation (heterosexual/homosexual) | .197 |
| Lifetime consumption of child abuse material (CSAM) or indicative pictures (yes/no) | .871 |
| Relative VRT index ^b^ | (.285/.016/.041) |

^b^ Relative VT index is obtained by subtracting the maximum reaction time of the preferred adult category (Tanner 4-5) from the maximum reaction time of the preferred child category (Tanner 1-3) for the sexual orientation-matched VT.
